# Development and comparative evaluation of LAMP, nested PCR and Real-time PCR assays for detecting *Fusarium tricinctum*, a fungal pathogen of *Zanthoxylum bungeanum*

**DOI:** 10.1186/s12866-025-04295-8

**Published:** 2025-08-30

**Authors:** Yuqing Dong, Jing Song, Shuying Li, Jiasui Zhan, Tianhui Zhu

**Affiliations:** 1 College of Forestry, Sichuan Agricultural University, Chengdu, 611130 Sichuan China; 2 Department of Forest Mycology and Plant Pathology, Swedish University of Agricultural Sciences, Uppsala, 75007 Sweden; 3 Forest Ecology and Conservation in the Upper Reaches of the Yangtze River Key Laboratory of Sichuan Province, Chengdu, 611130 Sichuan China; 4 Sichuan Mt. Emei Forest Ecosystem National Observation and Research Station, Chengdu, 611130 Sichuan China

**Keywords:** *Zanthoxylum bungeanum* gummosis, *Fusarium tricinctum*, Nested PCR, Real-time PCR, LAMP, Molecular detection

## Abstract

**Background:**

*Zanthoxylum bungeanum* is a highly valuable economic tree species in China, widely cultivated for its aromatic peel, medicinal properties, and industrial applications. In recent years, *Fusarium tricinctum*, a pathogen causing gummosis in *Z. bungeanum*, has severely impacted production in Sichuan and Gansu provinces. Early detection of this pathogen is challenging due to its prolonged incubation period and nonspecific symptoms, which often lead to significant crop losses and secondary infections by insects. Traditional methods of morphological identification are time-consuming and lack accuracy, necessitating the development of rapid molecular diagnostic tools.

**Results:**

In this study, we screened potential target sequences from *F. tricinctum* and developed three rapid detection methods—loop-mediated isothermal amplification (LAMP), nested PCR, and real-time fluorescent quantitative PCR (qPCR) based on the *CYP51C* gene. All three methods demonstrated high specificity and effectiveness for early diagnosis of gummosis in *Z. bungeanum*. qPCR exhibited the highest sensitivity, detecting DNA concentrations as low as 3.1 fg/µL, which was tenfold more sensitive than LAMP and nested PCR. Additionally, qPCR enabled absolute quantification of the pathogen. Nested PCR showed exceptional stability and reliability, while LAMP provided rapid, cost-effective, and visually interpretable results, making it ideal for field applications.

**Conclusions:**

These findings demonstrate the potential of molecular techniques to overcome traditional diagnostic limitations, providing practical solutions for early pathogen detection and sustainable disease management in *Z. bungeanum*. Among the methods, LAMP is optimal for field applications due to its simplicity, speed, and visual interpretation.

**Supplementary Information:**

The online version contains supplementary material available at 10.1186/s12866-025-04295-8.

## Background

*Zanthoxylum bungeanum*, a perennial spiny deciduous shrub belonging to the genus of *Zanthoxylum* L. (Rutaceae family), is one of the most important economic tree species in China [1]. With a long history of cultivation, *Z. bungeanum* has a wide range of applications. Its peel is widely used as an aromatic spice to enhance flavor and stimulate appetite while its seeds can be processed to produce Zanthoxylum oil [2], which serves as both edible and industrial purposes. Furthermore, *Z. bungeanum* contains numerous bioactive compounds with medicinal value [3, 4], including volatile oils, amides, alkaloids and coumarins. These components exhibit various pharmacological effects such as anti-inflammatory, antioxidant, anticancer, hypoglycemic, and antihypertensive properties [5–8].

However, with the continuous expansion of agricultural and forestry cultivation, the sustainability of this important tree species is increasingly threatened by *Fusarium tricinctum*, the primary causal agent of *Z. bungeanum* gummosis disease [9]. The disease is particularly challenging to manage due to its prolonged latent period, nonspecific early symptoms, and frequent association with secondary pest infestations [10].

Early and accurate diagnosis of diseases is crucial for minimizing forestry losses, and rigorous pathogen inspection and quarantine are essential to prevent large-scale outbreaks. Current diagnostic methods for the pathogen of *Z. bungeanum* gummosis disease primarily rely on traditional morphological identification and conventional PCR techniques [10]. However, these approaches suffer from significant limitations: morphological identification requires specialized expertise and often fails during early infection stages, while conventional PCR lacks the sensitivity needed for reliable early detection [11]. These diagnostic shortcomings frequently result in delayed interventions, allowing pathogen establishment and spread before symptoms become apparent [12]. Consequently, there is an urgent need for rapid, sensitive, and field-deployable detection methods to enable timely disease management.

With the rapidly development of molecular biology, molecular detection techniques have also become increasingly modernized. Among them, molecular diagnostic methods based on PCR [13] have been increasingly applied in the identification and diagnosis of fungi, and have become an important means to solve the related problems of existing morphological identification [14]. Rashmi et al. [15] designed specific primers for *P. striiformis* f. sp. *tritici* causing wheat stripe rust, optimized the reaction system, and established a LAMP detection method capable of rapid and sensitive identification of the pathogen. Mercado-Blanco et al. [16] employed nested PCR to detect two distinct pathogenic types (defoliating and nondefoliating) of *Verticillium dahliae* infecting olive trees. Sarah et al. [17] developed a nested PCR assay targeting the *TEF-1α* sequence of *Fusarium solani* f. sp. *piperis* in black pepper soil and roots, achieving a high sensitivity for genomic DNA detection, demonstrating potential for field diagnosis. Furthermore, real-time PCR has provided technical support and epidemiological research tools for studying spinach Fusarium wilt caused by *Fusarium oxysporum* [18], as well as pine shoot tip dieback and tree mortality infected by *Diplodia sapinea* [19]. Despite these technological advances, there are currently no established rapid and portable molecular methods specifically developed for detecting *F. tricinctum* in *Z. bungeanum*.

The selection of appropriate genetic targets is crucial for developing molecular detection assays. Ribosomal DNA in eukaryotic cells has emerged as a common target for the detection of fungal pathogens due to its slow evolutionary rate and extensive conserved regions [20–22]. Recent molecular studies have also identified other multiple genetic markers for *Fusarium* identification, including the internal transcription spacer (IGS) [23], translation extension factor-1α [24], β-tubulin [25], calmodulin [26], Sterol 14α-demethylase (*CYP51C*) [27, 28], RNA polymerase II large subunit (*RPB1*) [29, 30], and RNA polymerase II second largest subunit (*RPB2*) [31]. Among these, *CYP51C* is particularly valuable as a *Fusarium*-specific marker that can distinguish between the closely related species such as *F. avenaceum* and *F. tricinctum*, making it an excellent candidate for developing species-specific PCR assays [27].

In this study, we developed and evaluated three molecular detection methods—LAMP, nested PCR, and qPCR—for rapid identification of *F. tricinctum*, the causal agent of *Z. bungeanum* gummosis. Targeting the *Fusarium*-specific *CYP51C* gene, we systematically compared these methods in terms of sensitivity, specificity, detection speed, and operational feasibility to establish optimized diagnostic protocols. Our work represents the first comprehensive evaluation of molecular detection approaches for *F. tricinctum* in *Z. bungeanum* systems, with three key objectives: (1) to develop optimized protocols for each method, (2) to compare their analytical performance under standardized conditions, and (3) to validate their efficacy using field-collected samples. The resulting assays address critical gaps in *Z. bungeanum* disease management by enabling early pathogen detection and timely implementation of control measures.

## Methods

### Fungal strains, plant culture, and DNA extraction

*F. tricinctum* was isolated from *Z. bungeanum* branches exhibiting gummosis symptoms collected in Hanyuan County, Ya’an City, Sichuan Province. Briefly, systematic sampling was performed by excising 5 × 5 mm tissue sections at the disease-health junction of infected branches. Following surface sterilization with 3% NaClO and 75% ethanol (60 s each), samples were rinsed thrice with sterile distilled water. Surface moisture was removed using sterile filter paper before culturing on PDA medium for isolation and purification. To evaluate primer specificity, 14 fungal strains were selected, including pathogens associated with *Z. bungeanum* diseases and common environmental fungi (Table 1). All fungal strains were maintained in the Laboratory of Forest Pathology at Sichuan Agricultural University. The *Z. bungeanum* cultivar ‘Dahongpao’ was selected as the host material to validate the practical efficacy of the molecular detection system, which was grown in the greenhouse of the College of Forestry at Sichuan Agricultural University.

**Table 1:** Fungal strains used in this study with their respective host plants and isolation sources

Number	Species	Host plant	Site
1	*Fusarium proliferatum*	*Zanthoxylum armatum*	Root
2	*Fusarium solani*	*Zanthoxylum bungeanum*	Branch
3	*Fusarium solani*	*Zanthoxylum bungeanum*	Root
4	*Fusarium avenaceum*	*Zanthoxylum bungeanum*	Branch
5	*Fusarium fujikuroi*	*Juglans regia* L.	Branch
6	*Fusarium equiseti*	*Zanthoxylum bungeanum*	Root
7	*Fusarium oxysporum*	*Zanthoxylum bungeanum*	Root
8	*Fusarium tricinctum*	*Zanthoxylum bungeanum*	Branch
9	*Arthrinium phaeospermum*	*Bambusa pervariabilis* × *Dendrocalamopsis grandis*	Branch
10	*Neofusicoccum parvum*	*Juglans regia* L.	Branch
11	*Botryosphaeria dothidea*	*Juglans regia* L.	Branch
12	*Nigrospora oryzae*	*Citrus maxima* (Burm.) Merr.	Branch
13	*Alternaria* sp.	*Zanthoxylum bungeanum*	Branch
14	*Clonostachys rosea*	*Zanthoxylum bungeanum*	Branch
15	*Arthrinium arundinis*	*Bambusa pervariabilis* × *Dendrocalamopsis grandis*	Branch

The fungal isolates from Table 1 were inverted cultured on PDA plates at 20 °C for 5 days, and the resulting mycelia were collected for DNA extraction. Fungal genomic DNA was extracted using the Column Fungal DNAout 2.0 Kit (Tiandz, Beijing, China) following the manufacturer’s protocol. DNA quality and concentration were assessed through agarose gel electrophoresis and a Nanodrop One spectrophotometer (Thermo Fisher Scientific, Waltham, MA, USA), and the DNA was diluted to 50ng/uL in nuclease-free water and stored to −20 °C for later use.

### Primer design

Target sequences commonly used for *F. tricinctum* were retrieved and downloaded via the NCBI portal (www.ncbi.nlm.nih.gov). Sequence alignment analysis was performed on the downloaded *F. tricinctum* sequences using MEGA5 software [32], enabling the selection of highly specific partial sequences within the *CYP51C* gene (accession number: GU785057.1) of *F. tricinctum* as target regions. Specific primers for LAMP were designed using Primer Explorer V5 (Eiken Chemical Co., Ltd., Tokyo, Japan) following standard LAMP primer design principles [33].

For nested PCR and qPCR, a highly conserved 300–500 bp region within the *CYP51C* gene was targeted. Primers were designed using Primer Premier 5.0, and their specificity was confirmed using NCBI’s Primer-BLAST tool [34]. All primers were commercially synthesized by Tsingke Biotechnology Co., Ltd. (Beijing, China), with sequences provided in Table 2.

**Table 2:** Primer sequences designed for *Fusarium tricinctum* detection targeting the *CYP51C* gene

Molecular assay	Primer name	Sequence (5’−3’)	Length (bp)
LAMP	F3	TCAACCTTGCGCACAAGC	18
	B3	CGGCATGGGAGATTTGACTT	20
	FIP	TTCTCCCAGGTCAATGGCGCACCTGGTGCAAGAGCTGT	38
	BIP	CAAAAGTTGACGCTCAACGGGCGTGGATGGGACTGTGAAGAC	42
Nested PCR	CYP-4 F	TCATGGAGGAGTTGTTCAGCGATAT	25
	CYP-4R	CGCATAAGCAGAGTTCTCGCCTAC	24
	C4-10 F	ACCTGGGAGAATCTACACAAGTT	23
	C4-10R	ATACTGCGCATCCATAAGTGTCC	23
qPCR	CPF	CACAATGGCGCGATCTGAAG	20
	CPR	GGACTGCTCACAGACTTGCT	20

### LAMP detection and optimization

LAMP reactions [35] were performed on the fungal strains listed in Table 1, with each reaction containing 2 µL of template DNA. Reaction mixtures were prepared according to the compositions specified in Table S1. Amplification was carried out in a 65 °C water bath for 1 h, followed by enzyme inactivation at 80 °C for 20 min using Bst DNA polymerase (New England Biolabs, cat. no. M0275L).

To establish optimal LAMP conditions, several parameters were systematically tested. Primer ratios (inner: outer primers, 1:1–1:10) and concentrations [F3/B3 (0.1–0.6 µM); FIP/BIP concentrations determined from ratio optimization] were evaluated. Additionally, reagent concentrations—including Mg²⁺ (2.0–12.0 mM), dNTPs (0.8–1.8 mM), and betaine (0.4–0.9 M)—were optimized. Reaction conditions such as temperature (60 °C, 65 °C, 70 °C) and duration (30 min, 40 min, 50 min, − 60 min) were also assessed.

Following amplification, reaction products were mixed with 1 µL of hydroxy naphthol blue (HNB) dye to visually detect positive results based on color change [36]. Optimal reaction conditions were further confirmed through agarose gel electrophoresis and sequencing validation.

### Optimization of nested PCR detection

The nested PCR detection system was optimized through a two-round amplification process [37]. In the first round, amplification was performed using the CYP-4 F/R primer pair with the reaction system detailed in Table S2. The resulting PCR products were then diluted ten-fold and used as templates for the second round of amplification with the C4-10 F/R primer pair, using a similar reaction system. Double-distilled water (ddH₂O) served as the negative control for both rounds. The thermal cycling protocol consisted of an initial denaturation at 94 °C for 5 min, followed by 35 cycles of denaturation at 94 °C for 30 s, annealing at 57 °C for 30 s, and extension at 72 °C for 1 min, with a final extension at 72 °C for 5 min. Amplification products were analyzed by 1% agarose gel electrophoresis and verified through sequencing.

To establish optimal reaction conditions, several parameters were systematically evaluated. The annealing temperature was tested across a range of 50–65 °C to determine the most specific amplification conditions. The optimal template volume for the first-round reaction was determined by testing additions ranging from 0.5 to 2.0 µL, while the optimal template concentration for the second-round reaction was determined by evaluating 10- to 100-fold dilutions of the first-round products. Each optimization experiment was performed in triplicate to ensure reproducibility. The final optimized nested PCR system was then used to test genomic DNA from *F. tricinctum* and other test strains list in Table 1, with successful amplification evaluated through electrophoretic detection of target bands.

### Optimization of qPCR

Conventional PCR amplification (Table S3) was performed on *F. tricinctum* and other 14 tested fungal strains using the specifically designed primer pair CPF/CPR to evaluate primer specificity. The amplified products were purified and cloned into the pClone007 Versatile Simple Vector (Tsingke, Beijing, China), followed by sequencing verification of positive clones. For quantitative analysis, standard curves were generated using ten-fold serial dilutions of the positive plasmid DNA [38].

The qPCR reaction conditions were optimized through systematic testing of several parameters. The annealing temperature was evaluated across a range of 56–62 °C to determine the optimal amplification specificity. Template concentration was optimized by testing addition volumes from 0.5 to 3.0 µL, while primer concentration was assessed between 2.0 and 4.0 mM. Each reaction included ddH₂O as a negative control. The thermal cycling protocol comprised an initial denaturation at 95 °C for 30 s, followed by 40 cycles of denaturation at 95 °C for 10 s and combined annealing/extension at the optimized temperature for 30 s. Melting curve analysis was performed by gradually increasing the temperature from 65 °C to 95 °C at a rate of 0.5 °C every 5 s.

Using the optimized conditions, the plasmid standard dilutions were amplified in triplicate to construct a standard curve by plotting the logarithm of template concentration against mean Cq values. The final optimized qPCR system was then used to test genomic DNA from *F. tricinctum* and other test strains, with each sample run in triplicate alongside negative controls. The detection specificity and performance of the qPCR system were thoroughly evaluated through these validation experiments.

### Sensitivity testing

The diagnostic specificity of all three optimized detection methods (LAMP, nested PCR, and qPCR) was verified using genomic DNA from all fungal strains listed in Table 1. Sensitivity testing was performed using ten-fold serial dilutions of *F. tricinctum* genomic DNA, ranging from 3.1 ng/µL to 3.1 fg/µL (seven concentration gradients). Each detection method was used to amplify these dilutions, with all reactions performed in triplicate to ensure reproducibility.

For comparative analysis, the diluted *F. tricinctum* DNA was also tested using conventional PCR. The sensitivity thresholds of all four methods (conventional PCR, nested PCR, qPCR, and LAMP) were then compared. Detection limits were determined through electrophoretic analysis of amplification products for the PCR-based methods, while qPCR sensitivity was assessed based on Cq values and standard curve analysis. This comprehensive sensitivity evaluation provided a clear comparison of the detection capabilities of each method.

### Verification of LAMP, nested PCR, and qPCR

To validate the practical application of the developed detection methods, pathogenicity re-isolation experiments were conducted using both in vitro and in vivo cultures of *F. tricinctum*. For in vitro testing, 3-year-old *Z. bungeanum* branches were cut into 10 cm segments, surface-sterilized, and rinsed with sterile water. Inoculation points were selected 3 cm from each end of the branches, where *F. tricinctum* mycelial plugs were introduced using sterile needles, with sterile PDA plugs serving as negative controls. The inoculated branches were maintained in a growth chamber at 25 °C. For in vivo experiments, inoculation points were established at 10 cm and 20 cm above ground level on 3-year-old *Z. bungeanum* trees under natural conditions following surface sterilization.

Following five days incubation period, total DNA was extracted from the central region of each inoculation site on *Z. bungeanum* using the using the Universal Genomic DNA Extraction Kit (Solarbio, Beijing, China) according to the manufacturer’s protocol. The extracted DNA was subsequently diluted to 50 ng/µL using nuclease-free water (ddH₂O). The processed samples were then analyzed with three optimized detection systems. In LAMP and nested PCR assays, *F. tricinctum* genomic DNA served as the positive control, while qPCR utilized a positive recombinant plasmid standard. All reactions included ddH₂O as a negative control to ensure specificity and prevent false positives. This comprehensive verification approach confirmed the reliability of each detection method under both controlled and natural conditions.

### Isolation and identification of pathogens

Pathogen re-isolation was performed from inoculated plant samples using standard tissue isolation techniques [39]. Following surface sterilization, infected tissues were plated on PDA medium and incubated at 28 °C for five days. Hyphal tips from colony margins were subsequently transferred to fresh PDA plates for purification through successive subculturing. The purified isolates underwent both morphological [40] and molecular characterization to confirm their identity.

Molecular identification employed fungal universal primers targeting the ITS (ITS1/4) [41], translation elongation factor 1-α (*TEF1-α*; ef1/ef2) [42], and large subunit ribosomal RNA (LSU; LR0R/LR5) [43] regions (Table S4). PCR products were sequenced commercially, and the resulting sequences were analyzed using NCBI BLAST comparison with reference *F. tricinctum* sequences. Phylogenetic analysis [44] was conducted through concatenated alignment of ITS, *TEF1-α*, and LSU sequences, with reference strain accession numbers provided in Table S5. In brief, the ITS, *TEF1-α*, and LSU sequences were aligned using MEGA 5 software [32]. The aligned sequences were subsequently concatenated in PhyloSuite [45] based on its default parameters. Finally, a phylogenetic tree was constructed using the concatenated sequences via the maximum likelihood [44]. This multi-locus approach provided robust confirmation that re-isolated pathogens matched the original inoculated strains, validating the infection model and detection methods.

## Results

### Specificity and optimization of LAMP

**Fig. 1:**
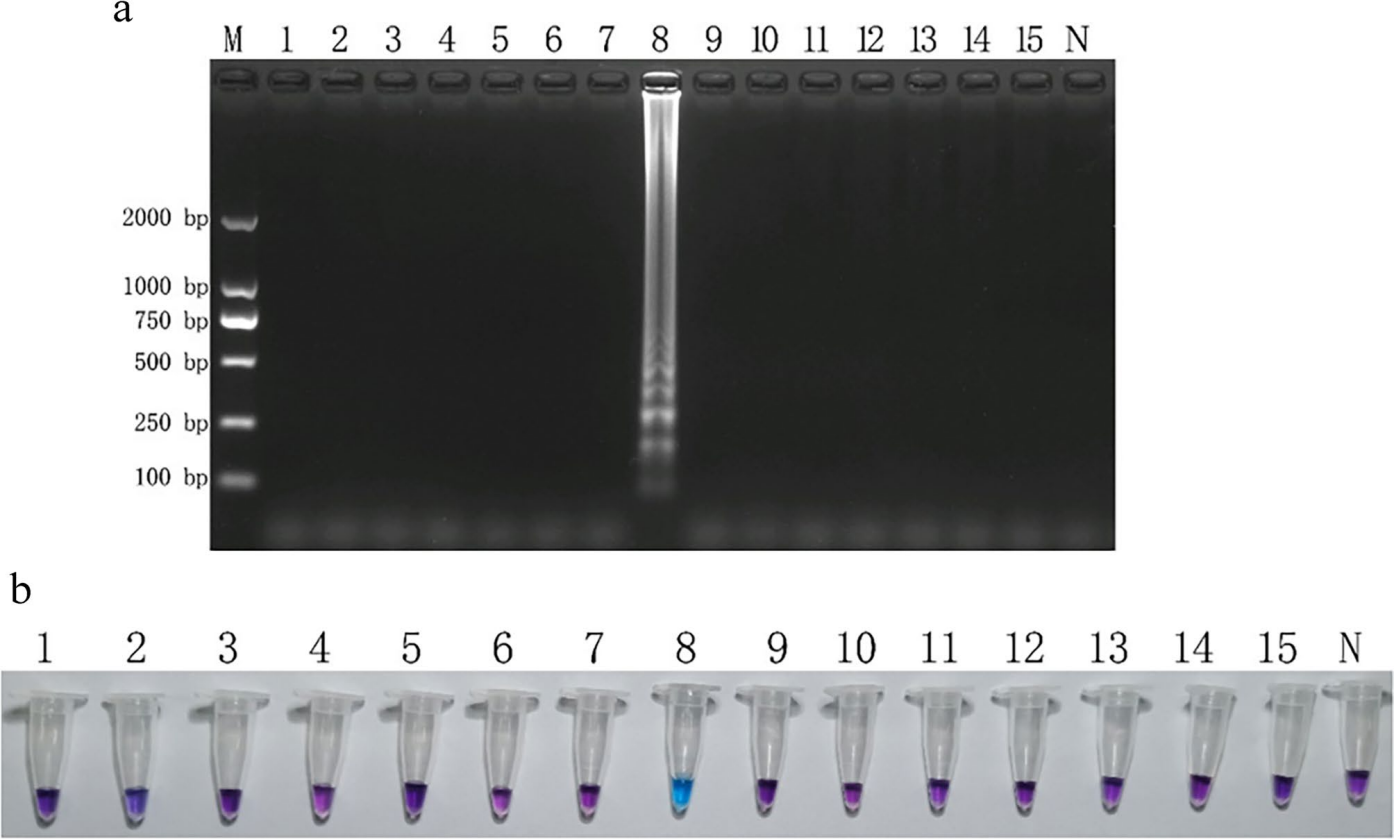
Specific detection of *Fusarium tricinctum* using loop-mediated isothermal amplification (LAMP). **a** Agarose gel electrophoresis showing characteristic ladder-like banding pattern of LAMP products (2% agarose). **b** Visual detection using hydroxy naphthol blue (HNB) dye, with color change from violet (negative) to sky blue (positive). M: DL2000 DNA marker; lanes 1–15: test strains corresponding to Table 1 (lane 8: *F. tricinctum* positive control); N: no-template negative control

The initial LAMP assay using primer sets F3/B3 and FIP/BIP demonstrated strong specificity when testing genomic DNA from all 15 strains, with amplification observed only in *F. tricinctum* (Figure S1). Through systematic optimization of reaction conditions, we determined the optimal parameters to be a 65 °C incubation for 50 min with an inner to outer primer ratio of 1:8 (0.8 µmol/L and 0.1 µmol/L, respectively). The optimized reagent concentrations included 8.0 mmol/L Mg²⁺, 1.6 mmol/L dNTPs, and 0.5 mol/L betaine. Electrophoretic analysis using this optimized system confirmed the exclusive amplification of *F. tricinctum* DNA, which produced the characteristic ladder-like banding pattern indicative of successful LAMP amplification (Fig. 1a). The specificity was further validated through the HNB colorimetric assay, where only reactions containing *F. tricinctum* DNA exhibited the distinctive color change from purple to blue (Fig. 1b). These results collectively demonstrate the high specificity and reliability of the optimized LAMP system for detecting *F. tricinctum*.

### Specificity and optimization of nested PCR

**Fig. 2:**
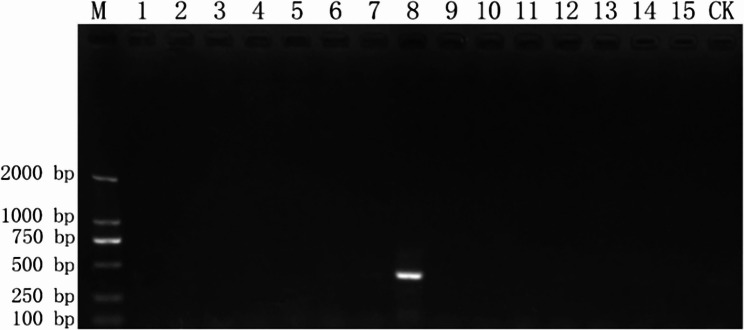
Specificity evaluation of nested PCR for *Fusarium tricinctum* detection. M: DL2000 DNA marker; lanes 1–15: test strains (lane 8: *F. tricinctum*); CK: negative control (ddH₂O)

The nested PCR assay showed excellent specificity during initial testing, with primers CYP-4 F/R and C4-10 F/R producing amplification only in *F. tricinctum* DNA, yielding distinct bands of approximately 600 bp and 400 bp (Figure S2). Sequencing analysis confirmed these products matched the target *CYP51C* gene sequence. Optimization experiments established the ideal first-round conditions as a 56 °C annealing temperature with 0.5 µL (15 ng) of template DNA, while the second round performed best at 53 °C using a 30-fold dilution of the first-round product. When applied to DNA from all test strains, the fully optimized nested PCR system specifically amplified only *F. tricinctum* DNA, generating a single bright band with no cross-reactivity observed (Fig. 2). These findings confirm the method’s diagnostic specificity for *F. tricinctum* detection.

### Construction of standard curve and specificity detection of qPCR

**Fig. 3:**
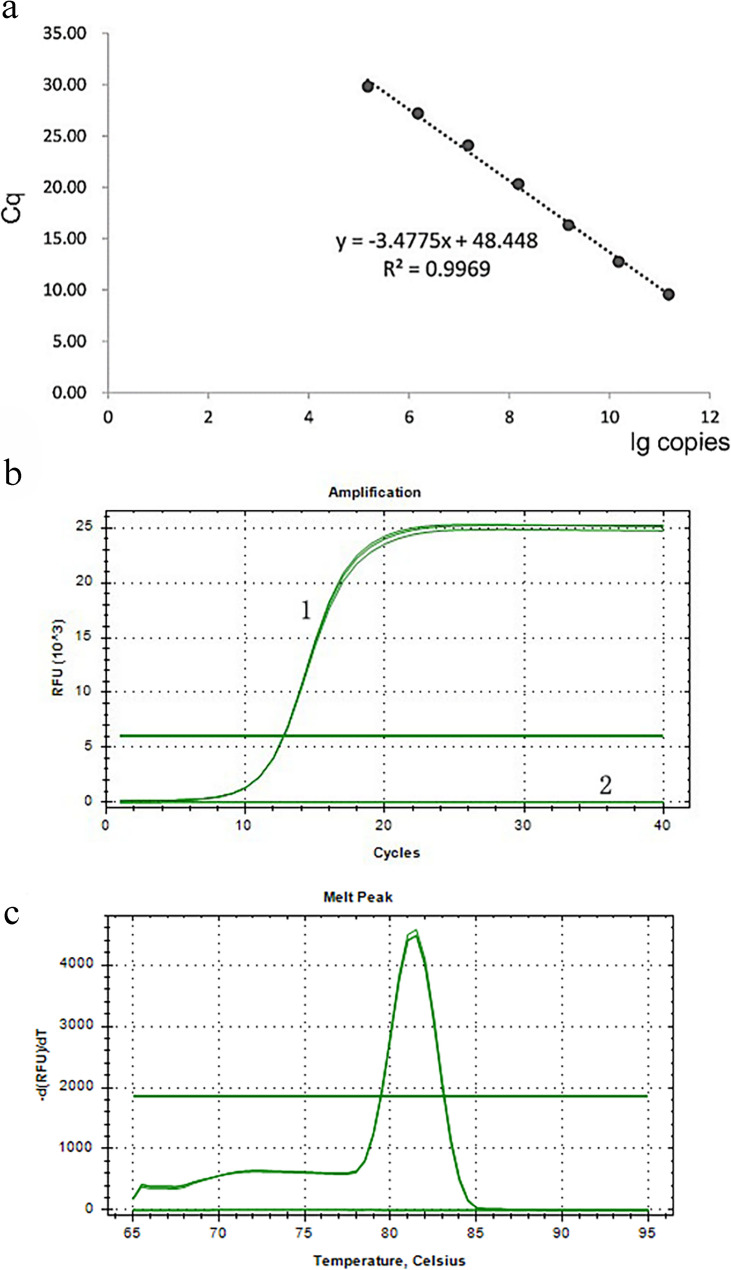
Quantitative real-time PCR analysis targeting the *CYP51C* gene of *Fusarium tricinctum*. **a** Standard curve generated from 10-fold serial dilutions of plasmid DNA (1.52 × 10¹⁰ to 1.52 × 10³ copies/µL; R² = 0.9969). **b **Amplification plots showing fluorescence intensity (ΔRn) versus cycle number.** c** Melting curve analysis (65–95 °C) demonstrating specific amplicon detection (single peak at 86.5 °C)

Initial specificity testing using CP-1 F/R primers in conventional PCR showed exclusive amplification of *F. tricinctum* DNA (Figure S3). The cloned target fragment with a concentration of 76 ng/µL was diluted to 2.5 ng/µL (1.52 × 10¹⁰ copies/µL) for standard curve generation. The resulting standard curve exhibited excellent linearity with a correlation coefficient (R²) of 0.9969 and a slope of −3.48, conforming to the equation y = −3.48x + 48.45 (Fig. 3a). After comprehensive optimization, the final 20 µL qPCR reaction system contained 1.0 µL of template DNA, 0.4 µL each of forward and reverse primers (10 µM), 10.0 µL of 2× SYBR qPCR Master Mix, and nuclease-free water to reach the final volume. Using the optimal annealing temperature of 58 °C, the assay specifically detected *F. tricinctum* DNA within 30 cycles, producing characteristic S-shaped amplification curves (Fig. 3b) and single-peak melting curves (Fig. 3c). No amplification was observed in reactions containing DNA from other test strains or the negative control, confirming the high specificity of the qPCR detection system for *F. tricinctum*.

### Sensitivity of optimized LAMP, nested PCR, and qPCR detection

The sensitivity of the three detection methods was evaluated using serial 10-fold dilutions of *F. tricinctum* genomic DNA, ranging from 31 ng/µL to 3.1 fg/µL (Table 3). Comparative analysis revealed qPCR as the most sensitive method, capable of detecting DNA concentrations as low as 3.1 fg/µL (Figure S6). Both LAMP (Figure S4) and nested PCR (Figure S5) demonstrated comparable sensitivity with detection limits of 31 fg/µL, while conventional PCR showed the lowest sensitivity at 3.1 pg/µL (Figures S5 and S6). When applied to inoculated *Z. bungeanum* branch samples, the methods maintained their relative sensitivity rankings. qPCR again showed superior performance by detecting *F. tricinctum* DNA at 320 fg/µL in plant samples, whereas LAMP and nested PCR achieved 3.2 pg/µL detection, and conventional PCR was limited to 3.2 ng/µL (Table 4). These results demonstrate the enhanced detection capability of the optimized systems, particularly qPCR, for identifying low pathogen concentrations in both purified DNA and complex plant samples.

**Table 3:** Sensitivity comparison of conventional PCR, LAMP, nested PCR, and qPCR using serial dilutions of *F. tricinctum* genomic DNA

	31ng/µL	3.1 ng/µL	310 pg/µL	31 pg/µL	3.1 pg/µL	310 fg/µL	31 fg/µL	3.1 fg/µL
PCR	+	+	+	+	+	-	-	-
LAMP	+	+	+	+	+	+	+	-
Nested PCR	+	+	+	+	+	+	+	-
qPCR	+	+	+	+	+	+	+	+

**Table 4:** Sensitivity comparison of detection methods for *F. tricinctum* in inoculated *Zanthoxylum bungeanum* samples

	32 ng/µL	3.2 ng/µL	320 pg/µL	32 pg/µL	3.2 pg/µL	320 fg/µL	32 fg/µL	3.2 fg/µL
PCR	+	+	-	-	-	-	-	-
LAMP	+	+	+	+	+	-	-	-
Nested PCR	+	+	+	+	+	-	-	-
qPCR	+	+	+	+	+	+	-	-

### Identification of pathogenic fungi in test samples

**Fig. 4:**
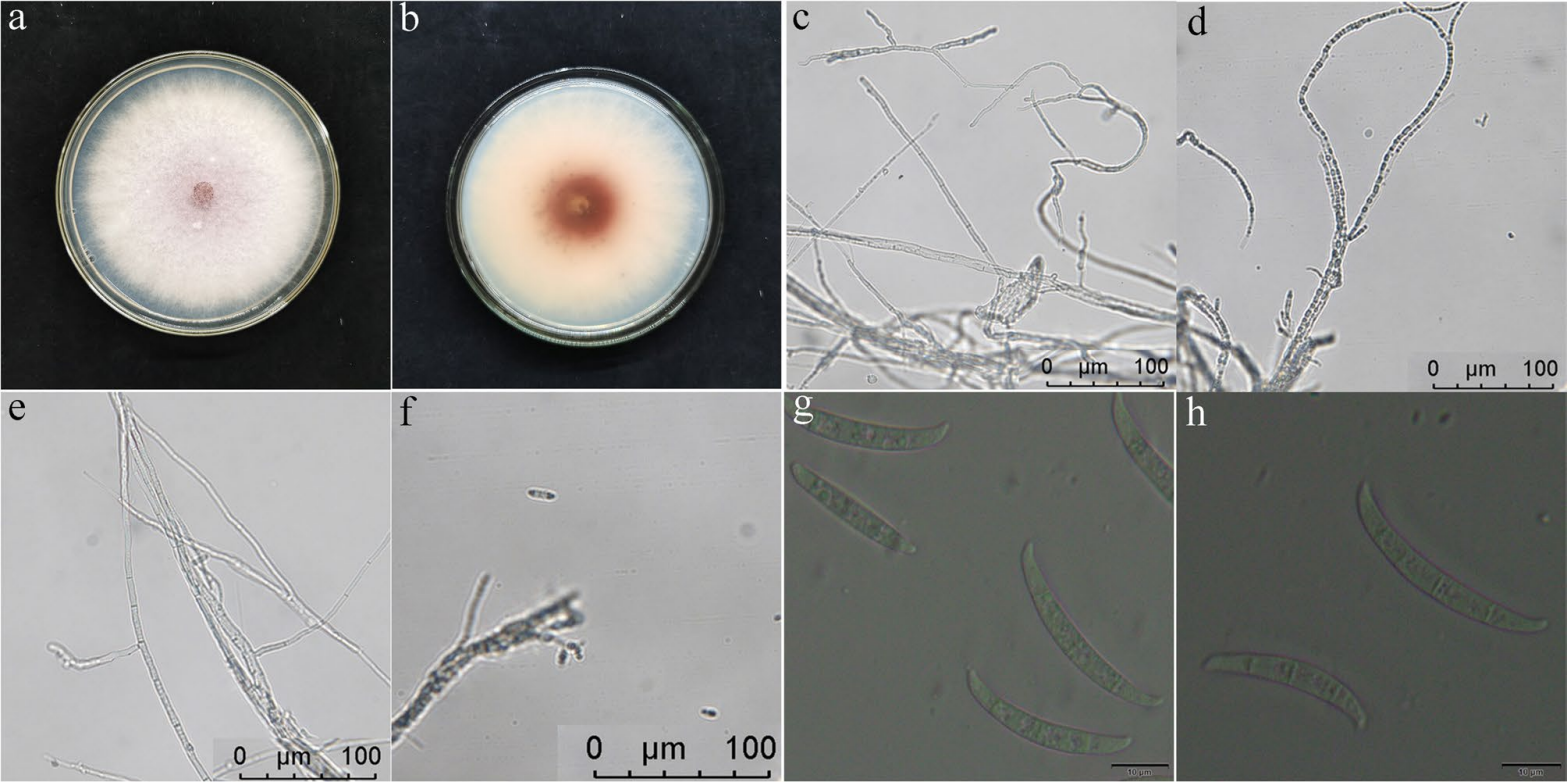
Morphological characterization of *Fusarium tricinctum* isolates. **a-d** Colony morphology on PDA medium after 5 d at 28 °C, showing (**a**) pale red aerial hyphae and (**b**) carmine pigmentation in basal regions. (**c-h**) Microscopic features: (**c-d**) septate hyphae, **(e-f**) conidiophores with phialides, and (**g-h**) characteristic falcate macroconidia (25.1–40.7 × 2.7–3.2 μm) produced in (**g**) PDA and (h) oat liquid cultures. Scale bars: 20 μm (**c-h**)

Morphological characterization of the dominant pathogen isolated from diseased *Z. bungeanum* revealed key features consistent with *F. tricinctum*. On PDA medium, colonies exhibited rapid growth with pale red aerial hyphae in central regions and white margins, accompanied by carmine pigment deposition (Figs. 4a-b). Microscopic examination showed septate hyphae producing characteristic conidiophores and large, curved macroconidia (25.1–40.7 × 2.7–3.2 μm) with 3–4 septa and distinctive spindle-shaped apical cells (Figs. 4c-g). Liquid culture in oat medium produced conidia morphologically identical to those from solid media (Fig. 4h), confirming stable growth characteristics across culture conditions.

**Fig. 5:**
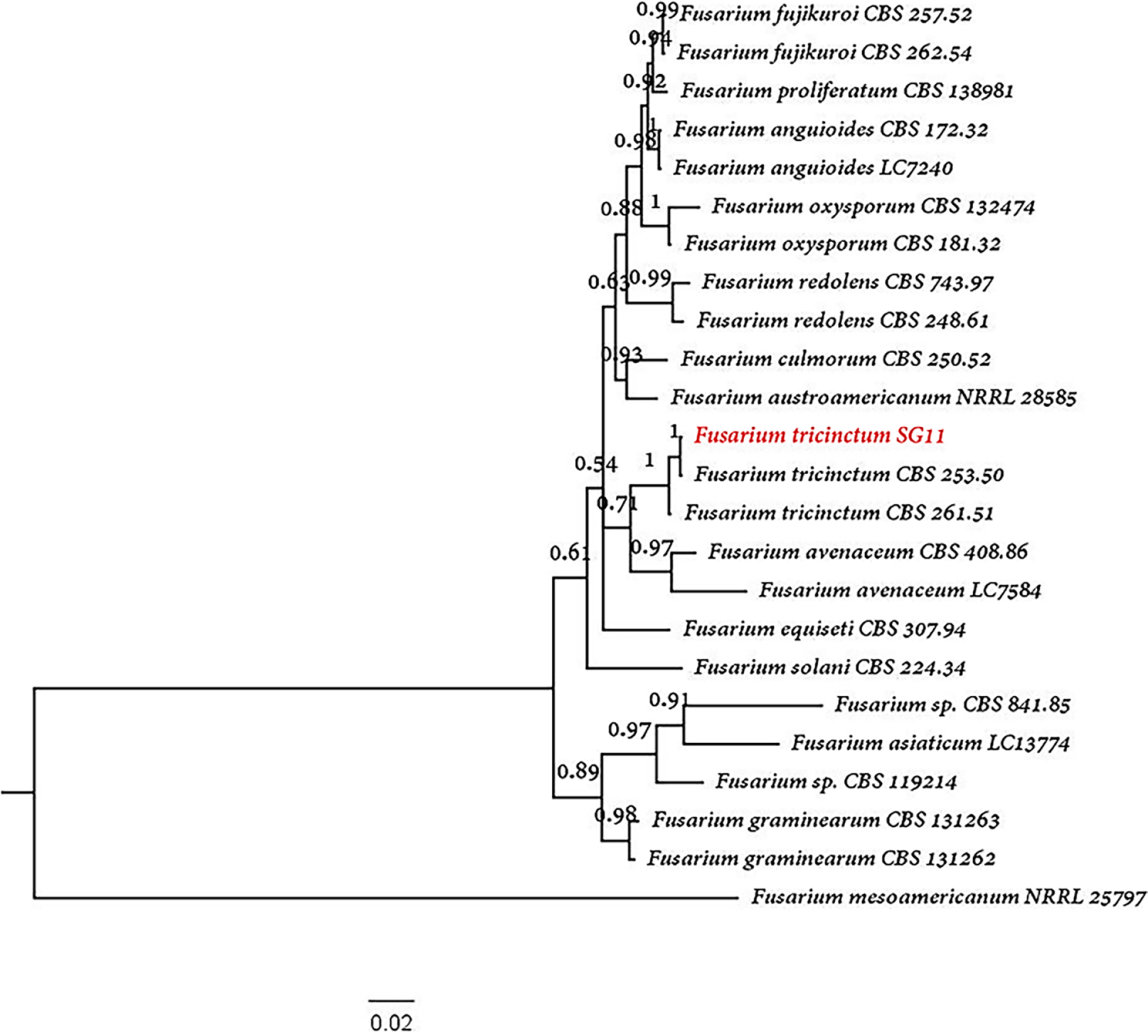
Phylogenetic analysis of *Fusarium tricinctum* strain SG11. Maximum likelihood tree based on concatenated sequences of ITS-rDNA, translation elongation factor 1-α (*TEF1-α*), and large subunit ribosomal RNA (*LSU*) genes (1,500 bootstrap replicates). The clinical isolate clusters with reference *F. tricinctum* strains (99.6% similarity), with *F. mesoamericanum* NRRL 25,797 as outgroup. Branch support values (> 70%) shown at nodes

Molecular analysis provided definitive identification through sequencing of ITS, TEF1-α, and *LSU* gene regions, showing 99.62%, 98.66%, and 99.44% similarity respectively to *F. tricinctum* reference sequences. Phylogenetic reconstruction using concatenated sequences placed the pathogen (strain SG11) within a well-supported (100% bootstrap) *F. tricinctum* clade, with *F. mesoamericanum* NRRL 25,797 as the outgroup (Fig. 5). The combined morphological and molecular evidence conclusively identified the isolated pathogen as *F. tricinctum*, matching the inoculated strain used in experimental infections.

## Discussion

The incubation period of *Z. bungeanum* gummosis is long, yet the disease progresses rapidly [46]. Traditional detection methods, such as morphological microscopy and PCR-based molecular techniques, are time-consuming [47], making rapid and efficient early monitoring of *F. tricinctum* particularly important. Previous studies have demonstrated that LAMP, qPCR, and nested PCR offer significantly higher sensitivity than conventional PCR, sometimes by a factor of 10 to 1000 [48, 49]. In this study, we screened potential target genes and identified *CYP51C* in *F. tricinctum* as the optimal sequence for designing specific primers. Using this gene, we successfully developed three molecular detection techniques—LAMP, nested PCR, and real-time fluorescence quantitative PCR—and validated their effectiveness through *Z. bungeanum* sample testing. The results confirmed that these methods are suitable for early gummosis diagnosis and hold strong potential for practical applications in production.

Recent advances in molecular biology have established LAMP as a powerful tool for detecting plant and animal pathogens [50], including bacteria, fungi, viruses, and nematodes [51–56]. In this study, we selected the *CYP51C* gene of *F. tricinctum* due to its high interspecific specificity. After designing and screening primers (F3/B3 and FIP/BIP), we optimized key reaction conditions, including primer concentration ratios, Mg²⁺ and dNTP concentrations, betaine concentration, reaction temperature, and duration. Detection results were visualized using agarose gel electrophoresis and hydroxy naphthol blue (HNB) staining. Specificity tests involving 15 strains and validation with *Z. bungeanum* samples confirmed the reliability of this detection system for early gummosis diagnosis, compared to existing the LAMP detection systems for *Fusarium* wilt of chickpea and *Astragalus membranaceus* root rot [57, 58], our system demonstrates superior sensitivity, cost efficiency, and practicality. The reaction can be performed under isothermal conditions, eliminating the need for expensive instruments, and yields results within 90 min. Additionally, the HNB colorimetric assay enables naked-eye interpretation without requiring gel electrophoresis. These advantages make our system highly suitable for field applications in *Z. bungeanum* production, underscoring its technical superiority and broad application potential.

Nested PCR involves two pairs of nested primers to enhance amplification, offering stronger specificity and higher sensitivity [59]. This dual-amplification approach prevents incorrect fragment amplification and reduces product contamination, making it particularly effective for detecting target sequences with high sensitivity and stability, especially in diseases with subtle symptoms. The technique has been widely adopted for detecting major forest diseases including eucalyptus blight [60], olive blight [16], and red band needle blight [61]. In this study, we designed two specific nested primer pairs (CYP-4 F/R and C4-10 F/R) targeting the *CYP51C* gene of *F. tricinctum*, which demonstrated dual specificity. Through optimization experiments, we determined the optimal annealing temperatures to be 56 °C for the outer primers and 53 °C for the inner primers. Combined with template concentration optimization, this nested PCR system produced clear, bright amplification bands, confirming its detection efficacy.

Real-time fluorescence quantitative PCR represents a significant advancement over traditional PCR by enabling quantitative analysis through fluorescent signal detection [62, 63]. This method overcomes the qualitative limitations of conventional PCR while offering superior specificity, exceptional sensitivity, real-time monitoring, and automated analysis [64, 65]. In our study, we developed highly specific primers (CP-1 F/R) for the *CYP51C* gene and optimized the reaction system using SYBR Green I fluorescent dye. Through careful optimization of annealing temperature, DNA template concentration, and primer concentration, we established a robust qPCR detection system. When testing target strain genomic DNA and *Z. bungeanum* samples, the system showed characteristic S-shaped amplification curves and single-peak melting curves, confirming specific amplification. With sensitivity tenfold higher than LAMP and nested PCR, this method provides laboratory-grade level support for the early warning of *Z. bungeanum* gummosis.

## Conclusion

In conclusion, the three molecular biology detection methods established in this address the limitations of traditional disease diagnosis approaches (time-consuming processes and uncertain accuracy) while providing effective tools for early diagnosis and prevention. Comparative analysis reveals that the nested PCR offers high stability but suffers from operational complexity and longer processing times, limiting its field applicability. While qPCR demonstrates unmatched sensitivity, its requirement for expensive instrumentation restricts its use to well-equipped laboratories. In contrast, the LAMP method combines high sensitivity, operational simplicity, rapid result, and visual interpretation. Therefore, the LAMP detection technology established in this study is the optimal choice for field detection of *F. tricinctum* in *Z. bungeanum* production and holds broad application prospects. making it the optimal choice for field detection of *F. tricinctum* in *Z. bungeanum* production with significant practical application potential.

## Supplementary Information

Supplementary Material 1.

Supplementary Material 2: Table S1. LAMP detection initial reaction system. Table S2. Nested PCR detection initial reaction system. The template for the first round was DNA, and the template for the second round was a tenfold dilution product of the first round of PCR. Table S3. Real Time-qPCR detection system. Table S4. Fungal universal primer sequences. Table S5. Information on the entry numbers of strains used for phylogenetic analysis. Figure S1. The results of LAMP amplification using specific primers. (a) the gel electrophoretogram. (b) Hydroxy naphthol blue (HNB) color rendering. M: D2000; 1-15: numbers as the strain numbers in Table 1, N: negative control. Figure S2. The schematic diagram of the result of nested PCR amplification using specific primers. (a) primer CYP-4F/R. (b) primer C4-10F/R. M: D2000; 1-15: numbers as the strain numbers in Table 1, CK: negative control. Figure S3. A schematic diagram of the result of general PCR amplification using specific primers CP-1F/R. M: D2000; 1-15: numbers as the strain numbers in Table 1, CK: negative control. Figure S4. Sensitivity of LAMP for detection of *F. tricinctum.* (a) the gel electrophoretogram. (b) Hydroxy naphthol blue (HNB) color rendering. M: D2000; 1-8: 31 ng/μL, 3.1 ng/μL, 310 pg/μL, 31 pg/μL, 3.1 pg/μL, 310 fg/μL, 31 fg/μL, 3.1 fg/μL; 9: Negative control. Figure S5. Sensitivity of nested PCR and general PCR for detection of *F. tricinctum.* (a) nested PCR. (b) general PCR. M: D2000; 1-8: 31 ng/μL, 3.1 ng/μL, 310 pg/μL, 31 pg/μL, 3.1 pg/μL, 310 fg/μL, 31 fg/μL, 3.1 fg/μL; 9: Negative control. Figure S6. Sensitivity of real time qPCR and general PCR for detection of *F. tricinctum.* (a) Amplification plots of RT-qPCR. (b) Melting curve of RT-qPCR. (c) general PCR. M: D2000; 1-8: 31 ng/μL, 3.1 ng/μL, 310 pg/μL, 31 pg/μL, 3.1 pg/μL, 310 fg/μL, 31 fg/μL, 3.1 fg/μL; CK: Negative control. 9: Negative control. 

## Data Availability

The datasets generated and/or analyzed during the current study are available in the NCBI repository (CYP-4: OQ184084; C4-10: OQ184078; CP-1: OQ184081; ITS: OQ152572; TEF1-α: OQ162039; LSU: OQ170961).

## Abbreviations

LAMP: Loop-mediated isothermal amplification
qPCR: Quantitative real-time PCR
CYP51C: Sterol 14α-demethylase
ITS: Internally transcribed spacer
TEF1-α: translation elongation factor 1-alpha
LSU: ribosomal large subunit
PDA: Potato dextrose agar (medium)
