# Supplementary material for: Development and comparative evaluation of LAMP, nested PCR and Real-time PCR assays for detecting Fusarium tricinctum, a fungal pathogen of Zanthoxylum bungeanum
Source: BMC Microbiol. 2025 Aug 30;25:568. doi: 10.1186/s12866-025-04295-8 (PMC12398972; doi:10.1186/s12866-025-04295-8)
Supplement: Supplementary file 1 — Supplementary Material 1. [file 12866_2025_4295_MOESM1_ESM.docx]

1. The original gel image of Figure S1a, the results of LAMP amplification using specific primers. M: D2000; 1-15: numbers as the strain numbers in Table 1, N: negative control.


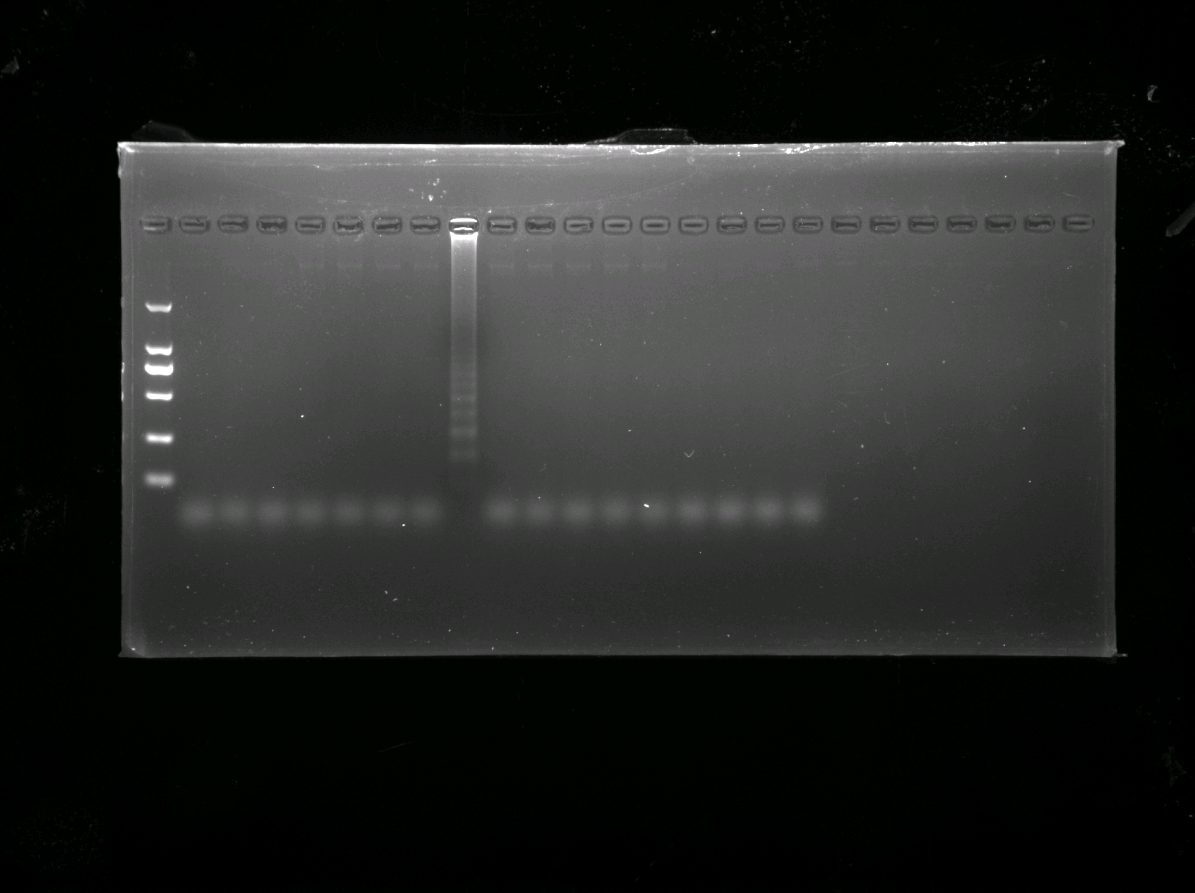


1

M

2

3

4

6

8

10

12

14

5

7

9

11

13

15

N

1. The original gel image of Figure 1a, agarose gel electrophoresis showing characteristic ladder-like banding pattern of LAMP products. M: DL2000 DNA marker; lanes 1-15: test strains corresponding to Table 1 (lane 8: *F. tricinctum* positive control); N: no-template negative control.
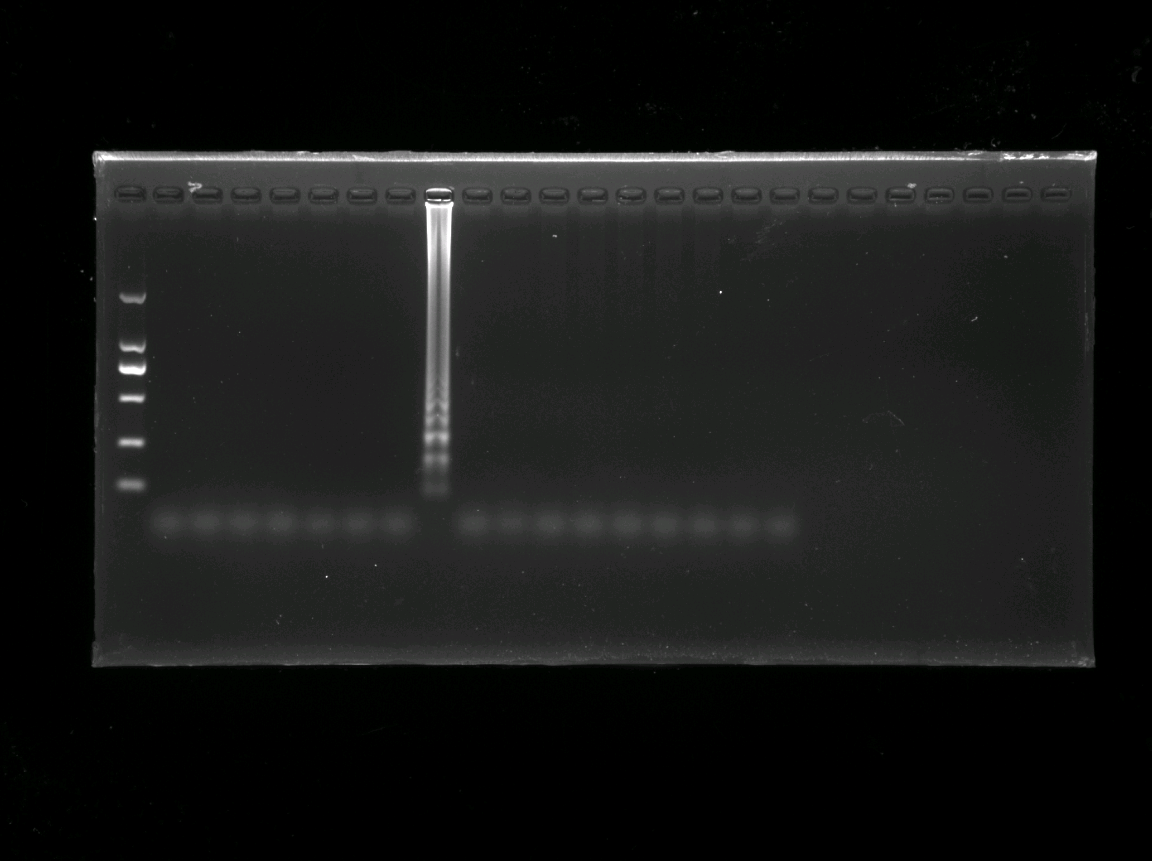


1

M

2

3

4

6

8

10

12

14

5

7

9

11

13

15

N

1. The original gel image of Figure S2, the schematic diagram of the result of nested PCR amplification using specific primers. (a) primer CYP-4F/R.


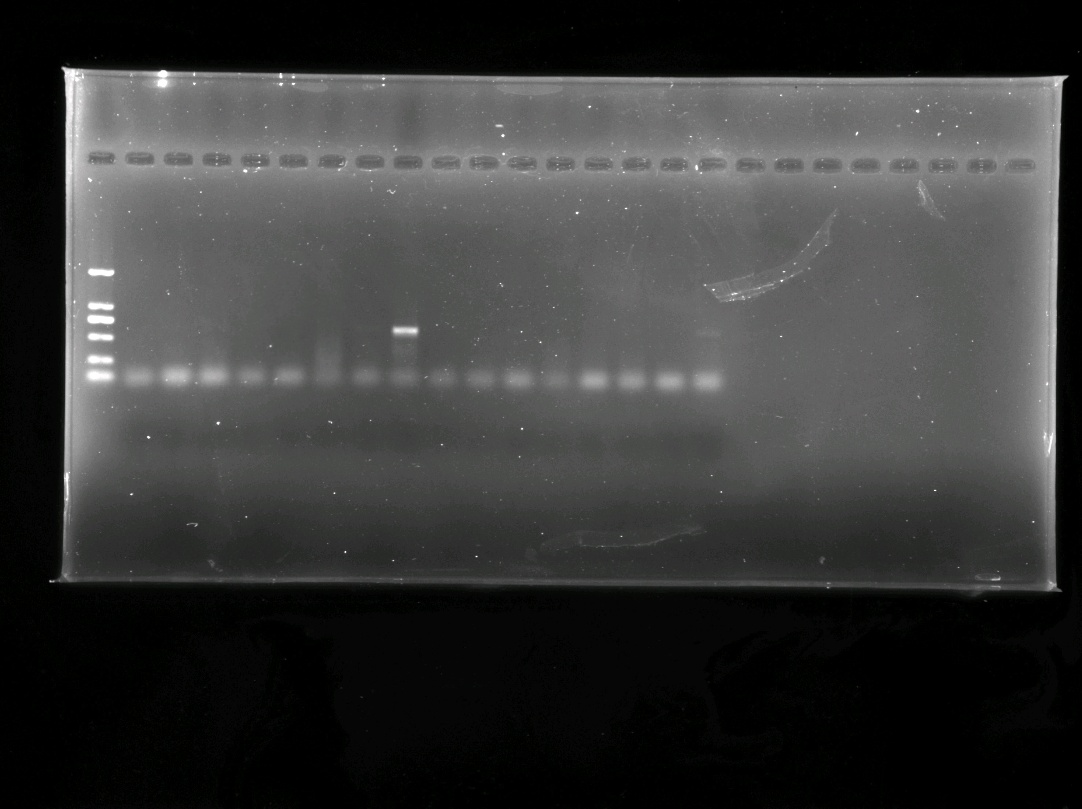


1

M

2

3

4

6

8

10

12

14

5

7

9

11

13

15

CK

(b) primer C4-10F/R. M: D2000; 1-15: numbers as the strain numbers in Table 1, CK: negative control.


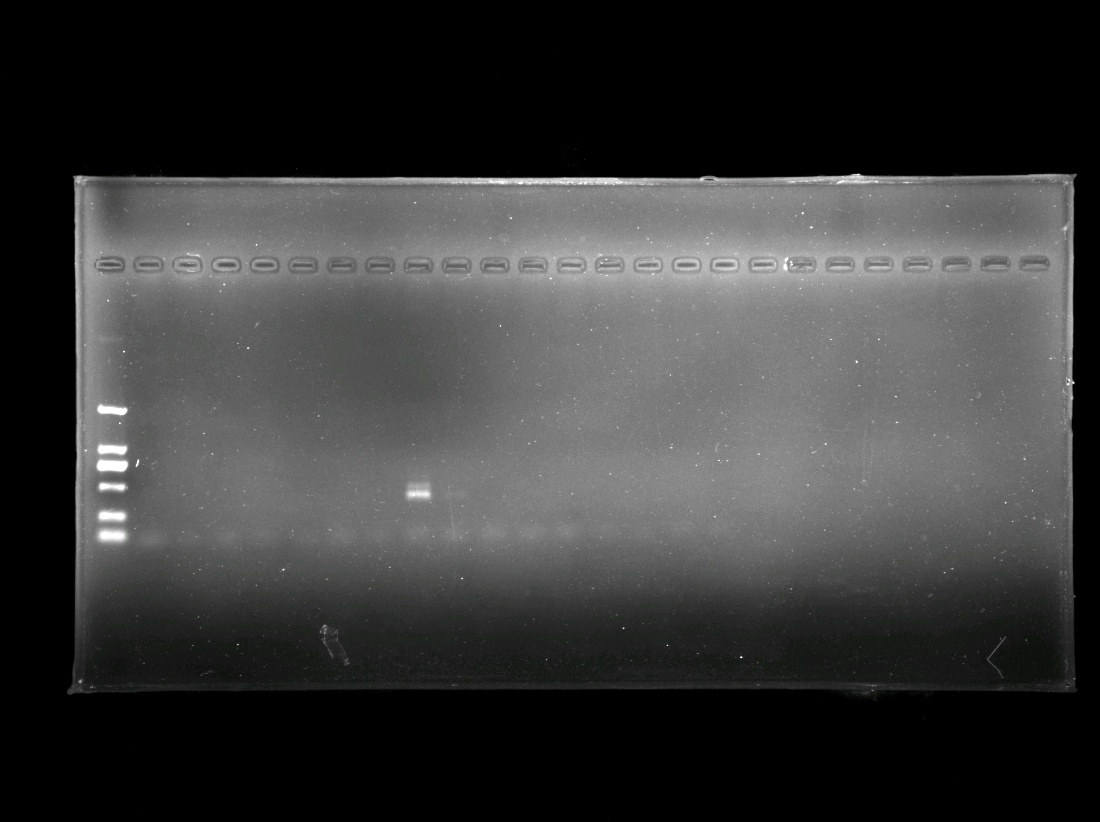


1

M

2

3

4

6

8

10

12

14

5

7

9

11

13

15

CK

1. The original gel image of Figure 2, Specificity evaluation of nested PCR for *Fusarium tricinctum* detection. M: DL2000 DNA marker; lanes 1-15: test strains (lane 8: *F. tricinctum*); CK: negative control (ddH₂O).


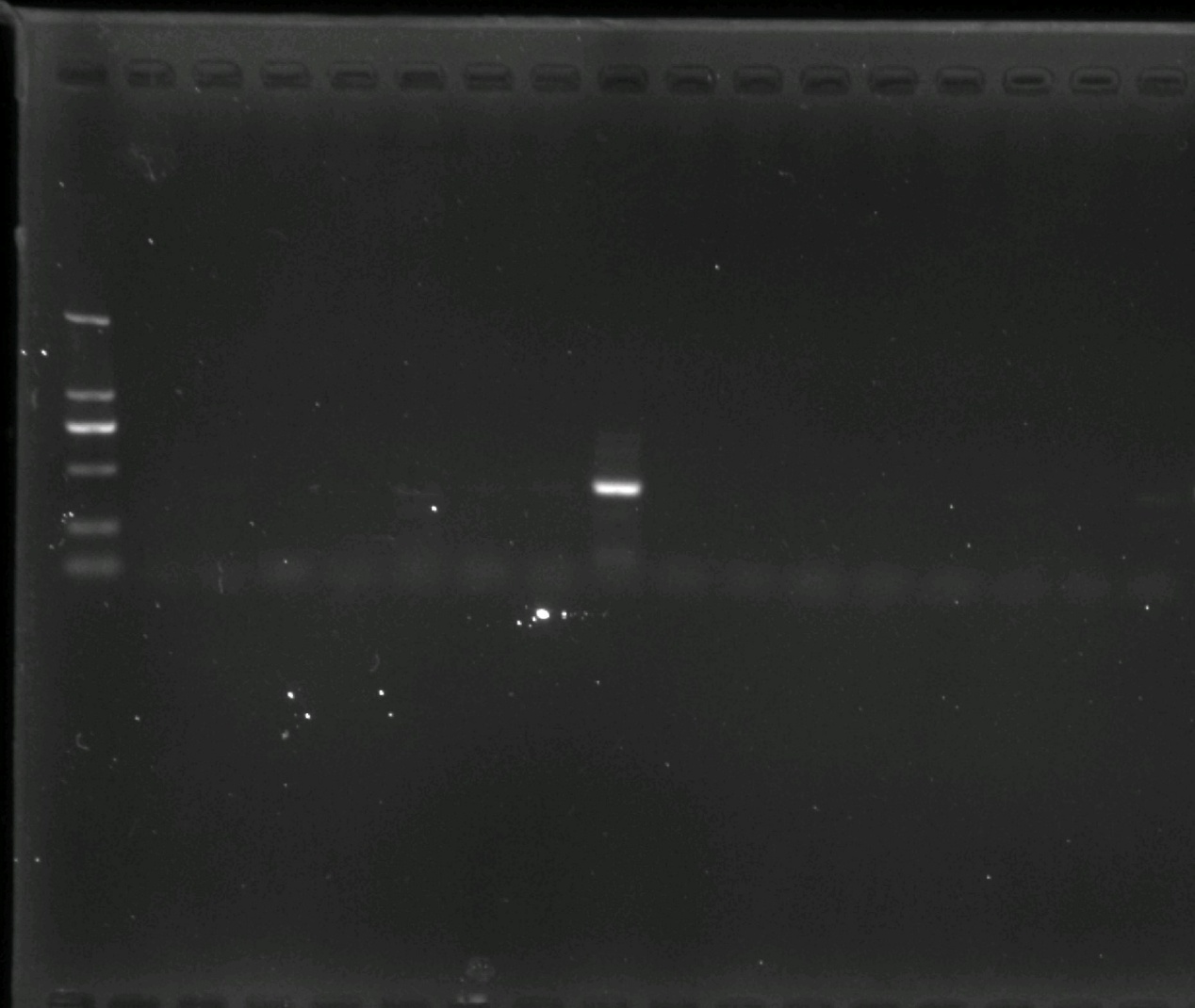


1

M

2

3

4

6

8

10

12

14

5

7

9

11

13

15

CK

1. The original gel image of Figure S3, a schematic diagram of the result of general PCR amplification using specific primers CP-1F/ R. M: D2000; 1-15: numbers as the strain numbers in Table 1, CK: negative control.


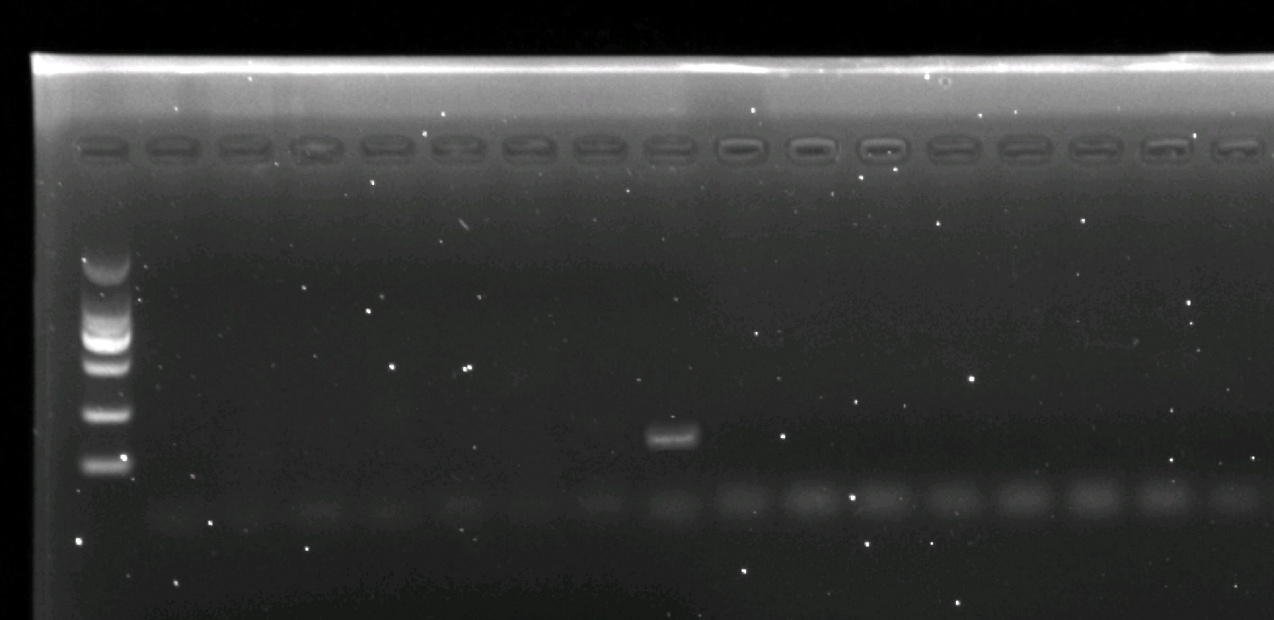


1

M

2

3

4

6

8

10

12

14

5

7

9

11

13

15

CK

1. The original gel image of Figure S4a, sensitivity of LAMP for detection of *F. tricinctum.* M: D2000; 1-8: 31 ng/μL, 3.1 ng/μL, 310 pg/μL, 31 pg/μL, 3.1 pg/μL, 310 fg/μL, 31 fg/μL, 3.1 fg/μL; 9: Negative control.


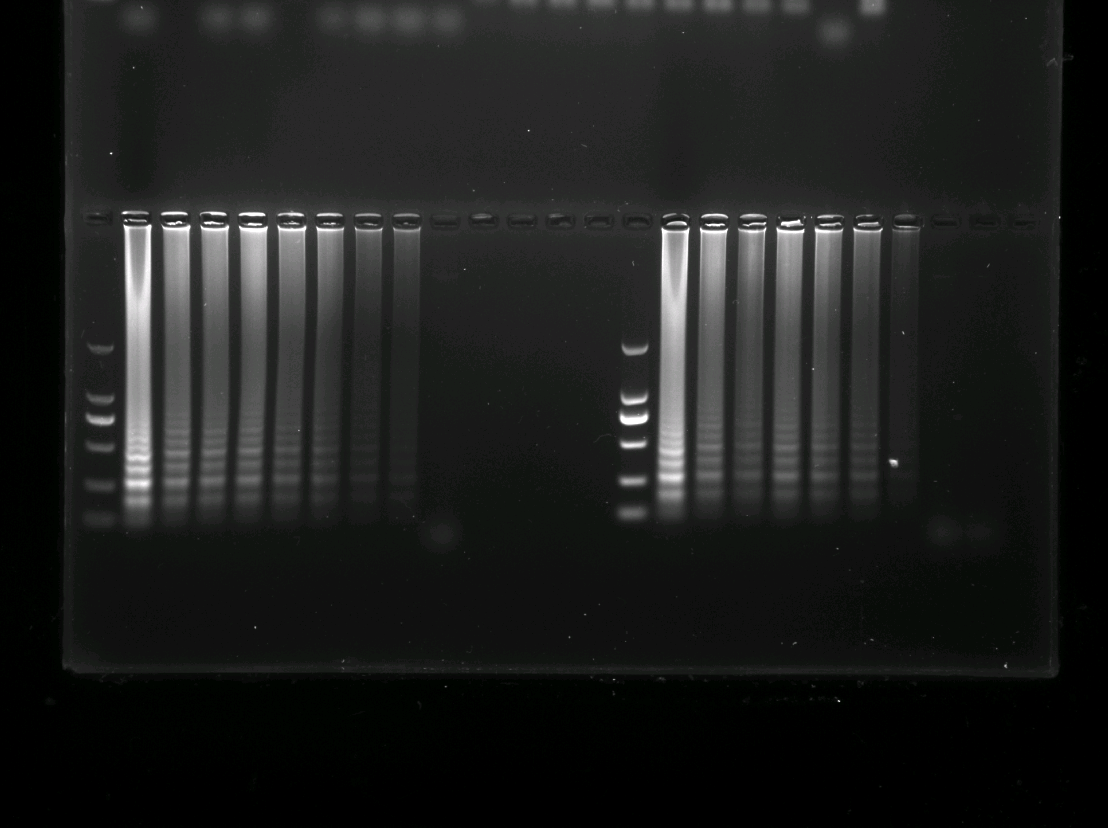


1

M

2

3

4

6

8

5

7

9

1. The original gel image of Figure S5, Sensitivity of nested PCR and general PCR for detection of F. tricinctum. (a) nested PCR.


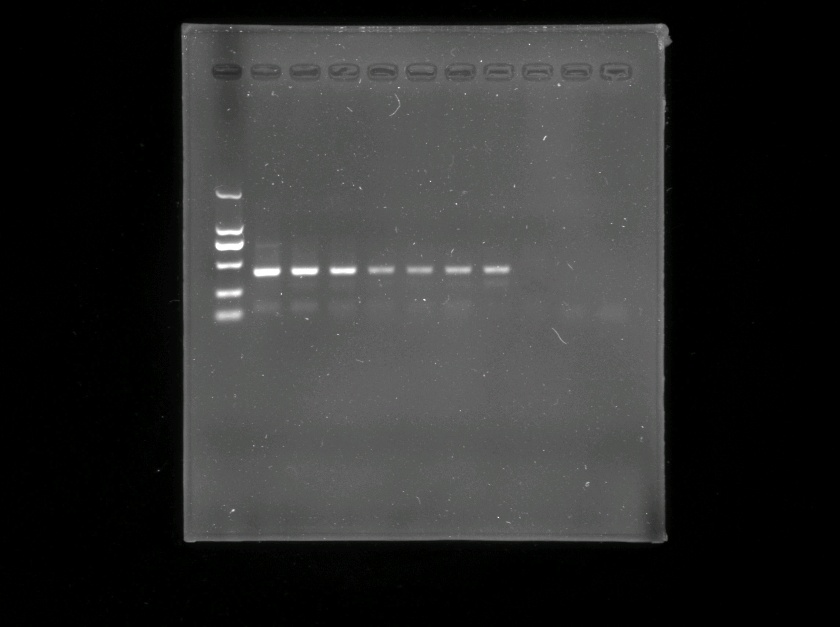


1

M

2

3

4

6

8

5

7

9

(b) general PCR. M: D2000; 1-8: 31 ng/μL, 3.1 ng/μL, 310 pg/μL, 31 pg/μL, 3.1 pg/μL, 310 fg/μL, 31 fg/μL, 3.1 fg/μL; 9: Negative control.


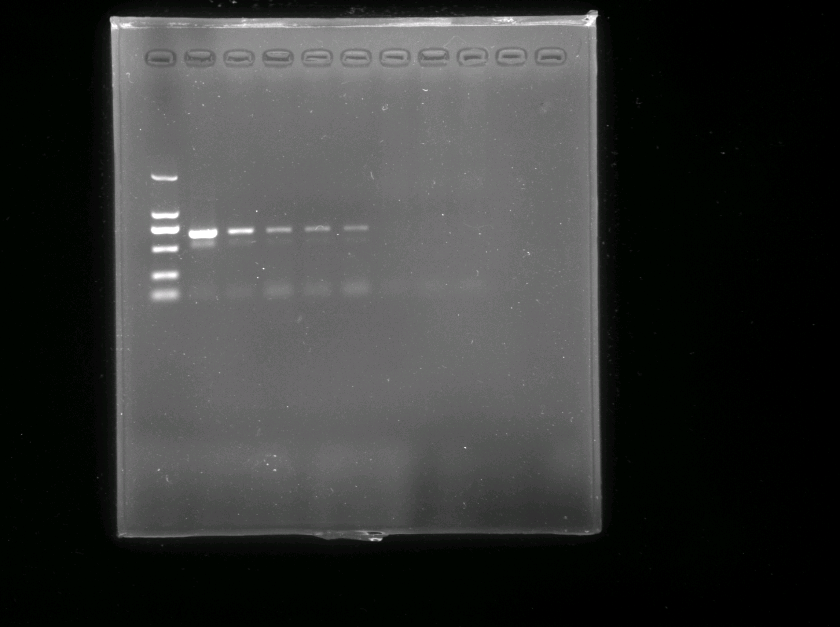


1

M

2

3

4

6

8

5

7

9

1. The original gel image of Figure S6c, sensitivity of general PCR. M: D2000; 1-8: 31 ng/μL, 3.1 ng/μL, 310 pg/μL, 31 pg/μL, 3.1 pg/μL, 310 fg/μL, 31 fg/μL, 3.1 fg/μL; 9: Negative control.


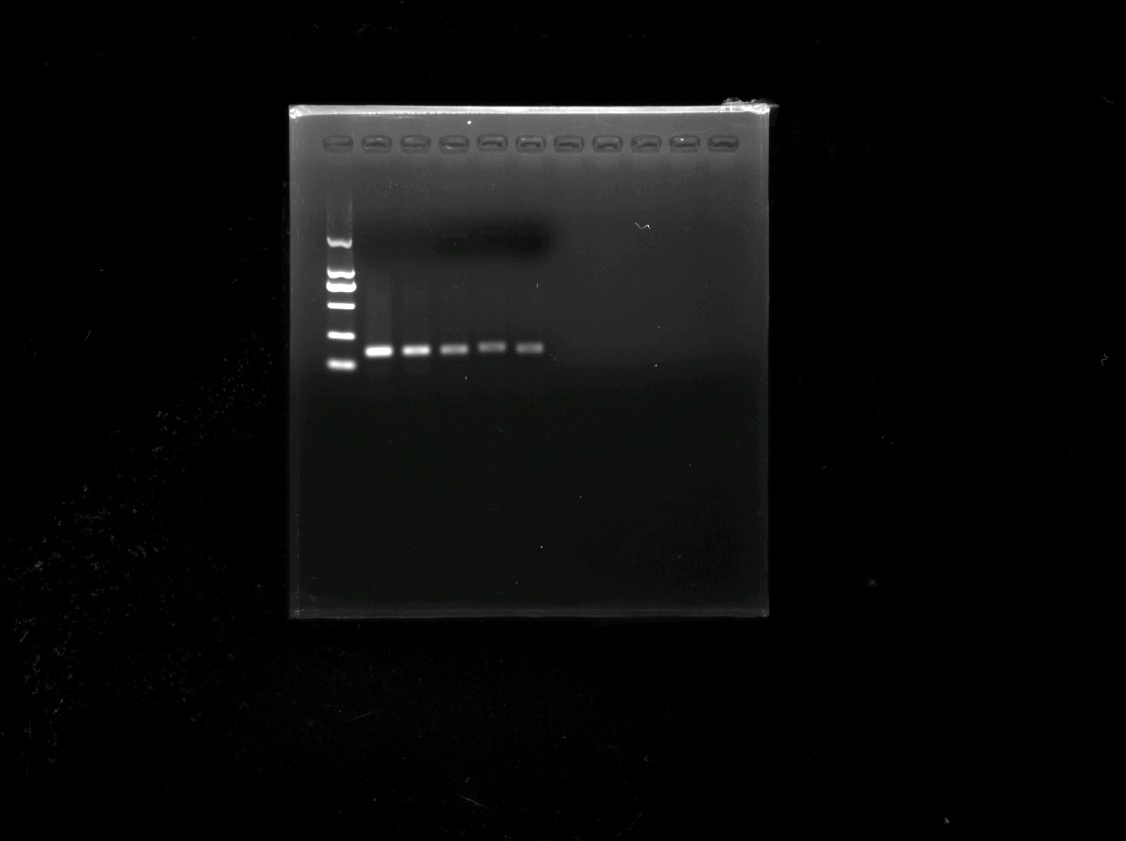


1

M

2

3

4

6

8

5

7

9
