# Supplementary material for: Development and comparative evaluation of LAMP, nested PCR and Real-time PCR assays for detecting Fusarium tricinctum, a fungal pathogen of Zanthoxylum bungeanum
Source: BMC Microbiol. 2025 Aug 30;25:568. doi: 10.1186/s12866-025-04295-8 (PMC12398972; doi:10.1186/s12866-025-04295-8)

| Component | Sample addition quantity (μL) | Final concentration (μmol/L) |
| --- | --- | --- |
| F3 (10 μmol/L) | 1 | 0.4 μmol/L |
| B3 (10 μmol/L) | 1 | 0.4 μmol/L |
| FIP (10 μmol/L) | 4 | 0.8 μmol/L |
| BIP (10 μmol/L) | 4 | 0.8 μmol/L |
| dNTPs (10 mmol/L) | 3.5 | 1.4 mmol/L each |
| MgSO_4_ (100 mmol/L) | 2.5 | 10.0 mmol/L |
| Betaine (5 mol/L) | 3 | 0.6 mol/L |
| Bst DNA Polymerase (8 U/μL) | 1 | 320 U/mL |
| DNA | 2 | 100 ng |
| 10×Thermolpol Buffer | 2.5 | 1× |
| ddH_2_O | 0.5 | Add to 25 μL |

**Table S1.** LAMP detection initial reaction system.

**Table S2.** Nested PCR detection initial reaction system. The template for the first round was DNA, and the template for the second round was a tenfold dilution product of the first round of PCR.

| Component | Sample addition quantity (μL) |
| --- | --- |
| Template | 1.0 |
| Forward Primer (10 μM) | 1.0 |
| Reverse Primer (10 μM) | 1.0 |
| 2×EasyTaq PCR SuperMix | 12.0 |
| ddH_2_O | 10.0 |
| Total | 25.0 |

**Table S3**. Real Time-qPCR detection system.

| Component | Sample addition quantity (μL) |
| --- | --- |
| DNA template | 0.5-4.0 |
| Forward Primer (10 μM) | 0.2-0.4 |
| Reverse Primer (10 μM) | 0.2-0.4 |
| 2×TransTaq High Fidelity (HiFi) PCR SuperMix II (-dye) | 12.5 |
| Nuclease-free Water | - |
| Total | 20.0 |

**Table S4.** Fungal universal primer sequences

| Primer name | Sequence (5’-3’) | Size (bp) |
| --- | --- | --- |
| ITS1 | TCCGTAGGTGAACCTGCGG | 19 |
| ITS4 | TCCTCCGCTTATTGATATGC | 20 |
| ef1 | ATGGGTAAGGA（A/G）GACAAGAC | 20 |
| ef2 | GGA（G/A）GTACCAGT（G/C）ATCATGTT | 21 |
| LR0R | GTACCCGCTGAACTTAAGC | 19 |
| LR5 | ATCCTGAGGGAAACTTC | 17 |

**Table S5**. Information on the entry numbers of strains used for phylogenetic analysis

| Species name | Strain No. | GenBank | | |
| --- | --- | --- | --- | --- |
|  |  | ITS | *TEF1-α* | *LSU* |
| *Fusarium* sp*.* | CBS 841.85 | MH861917 | NA | MH873606 |
| *Fusarium* sp*.* | CBS 119214 | EU552132 | EU552097 | NA |
| *Fusarium asiaticum* | LC13774 | MW016610 | MW620071 | MW474596 |
| *Fusarium graminearum* | CBS 131263 | JX162377 | JX118987 | MH877365 |
| *F. graminearum* | CBS 131262 | JX162364 | JX118973 | MH877364 |
| *Fusarium solani* | CBS 224.34 | MH855493 | JX435169 | MH866985 |
| *Fusarium culmorum* | CBS 250.52 | MH857016 | DQ453702 | MH868545 |
| *Fusarium austroamericanum* | NRRL 28585 | AY452872 | MW233095 | NA |
| *Fusarium redolens* | CBS 743.97 | AB304483 | MT010987 | NA |
| *Fusarium redolens* | CBS 248.61 | MH858041 | NA | MH869606 |
| *Fusarium anguioides* | CBS 172.32 | MH855263 | NA | MH866715 |
| *F. anguioides* | LC7240 | MW016402 | MW580442 | MW474388 |
| *Fusarium fujikuroi* | CBS 257.52 | MH857023 | MW402119 | MH868552 |
| *F. fujikuroi* | CBS 262.54 | MH857321 | MW402120 | NA |
| *Fusarium proliferatum* | CBS 138981 | KT716199 | KT716210 | KT716199 |
| *Fusarium oxysporum* | CBS 132474 | MH866022 | MH485020 | MH877470 |
| *F. oxysporum* | CBS 181.32 | MH855270 | MH484958 | MH866721 |
| *F. equiseti* | CBS 307.94 | MH862468 | KR071777 | MH874117 |
| *Fusarium tricinctum* | CBS 261.51 | MH856847 | DQ531562 | MH868367 |
| *F. tricinctum* | CBS 253.50 | KR071697 | KR071775 | MH868113 |
| *Fusarium avenaceum* | CBS 408.86 | MH864972 | MW928836 | MH876425 |
| *F. avenaceum* | LC7584 | MW016674 | MW620135 | MW474660 |
| *Fusarium mesoamericanum* | NRRL 25797 | AF006344 | MW233083 | AF006324 |

NA: The data does not exist.

**Figure S1**. The results of LAMP amplification using specific primers. (a) the gel electrophoretogram. (b) Hydroxy naphthol blue (HNB) color rendering. M: D2000; 1-15: numbers as the strain numbers in Table 1, N: negative control.


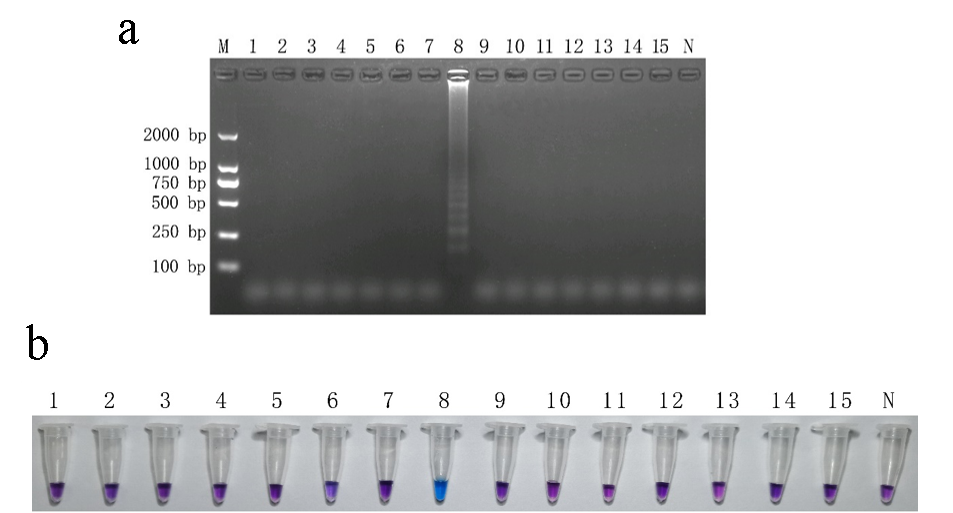


**Figure S2**. The schematic diagram of the result of nested PCR amplification using specific primers. (a) primer CYP-4F/R. (b) primer C4-10F/R. M: D2000; 1-15: numbers as the strain numbers in Table 1, CK: negative control.


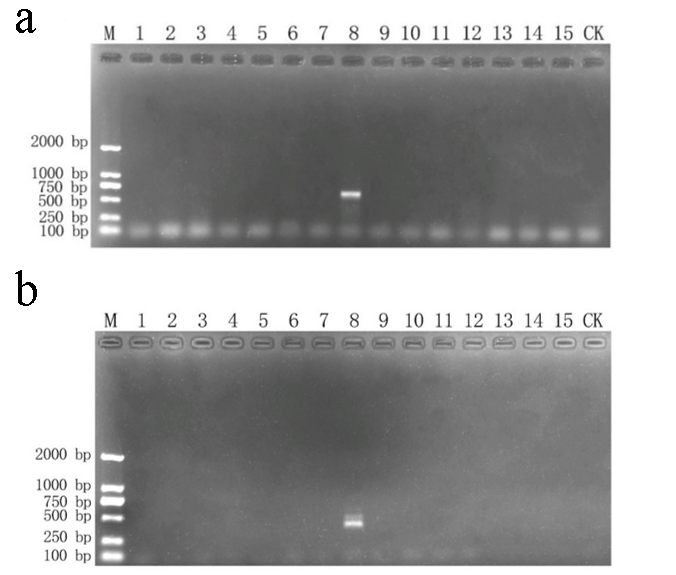


**Figure S3**. A schematic diagram of the result of general PCR amplification using specific primers CP-1F/ R. M: D2000; 1-15: numbers as the strain numbers in Table 1, CK: negative control.


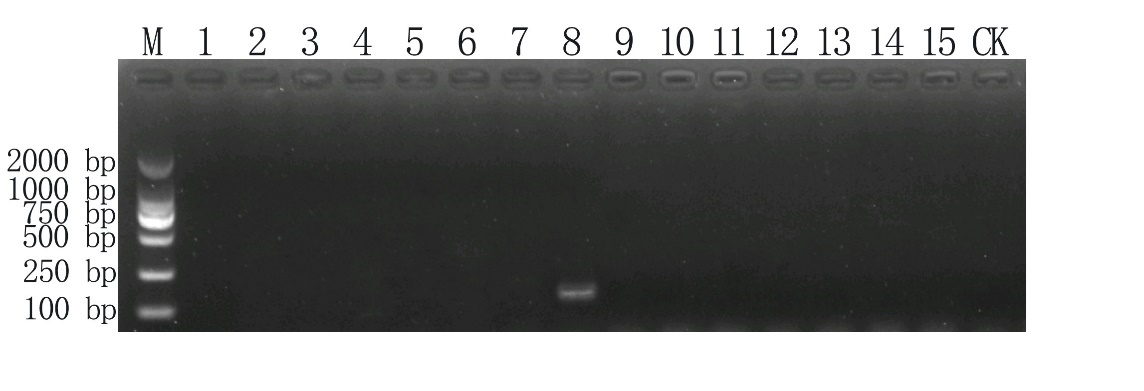


**Figure S4**. Sensitivity of LAMP for detection of *F. tricinctum.* (a) the gel electrophoretogram. (b) Hydroxy naphthol blue (HNB) color rendering. M: D2000; 1-8: 31 ng/μL, 3.1 ng/μL, 310 pg/μL, 31 pg/μL, 3.1 pg/μL, 310 fg/μL, 31 fg/μL, 3.1 fg/μL; 9: Negative control.


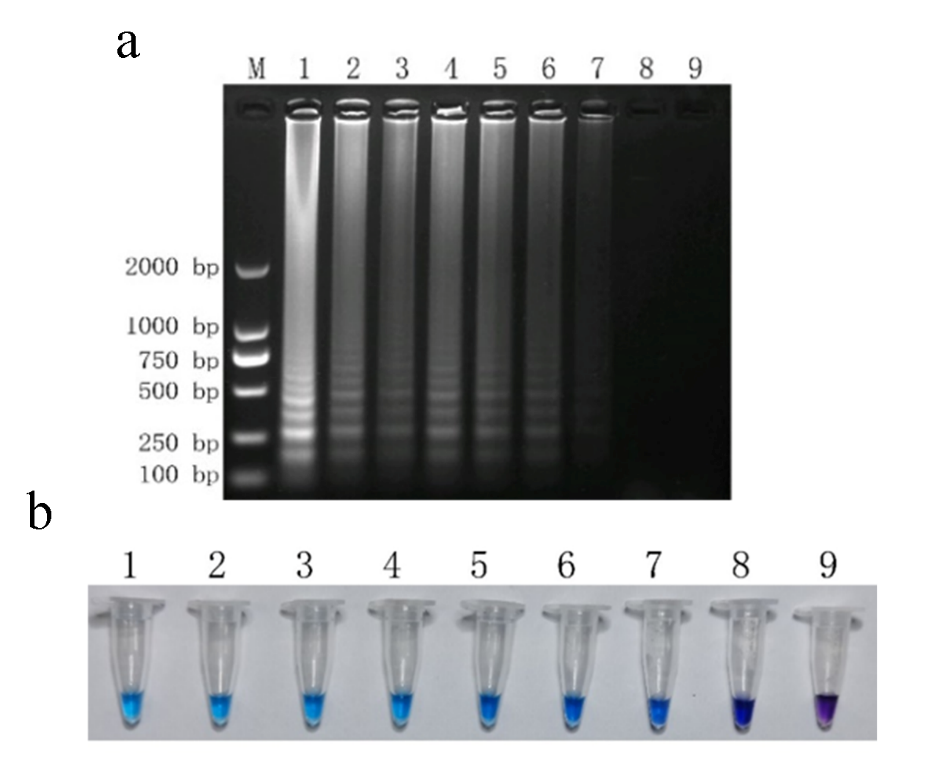


**Figure S5**. Sensitivity of nested PCR and general PCR for detection of *F. tricinctum.* (a) nested PCR. (b) general PCR. M: D2000; 1-8: 31 ng/μL, 3.1 ng/μL, 310 pg/μL, 31 pg/μL, 3.1 pg/μL, 310 fg/μL, 31 fg/μL, 3.1 fg/μL; 9: Negative control.


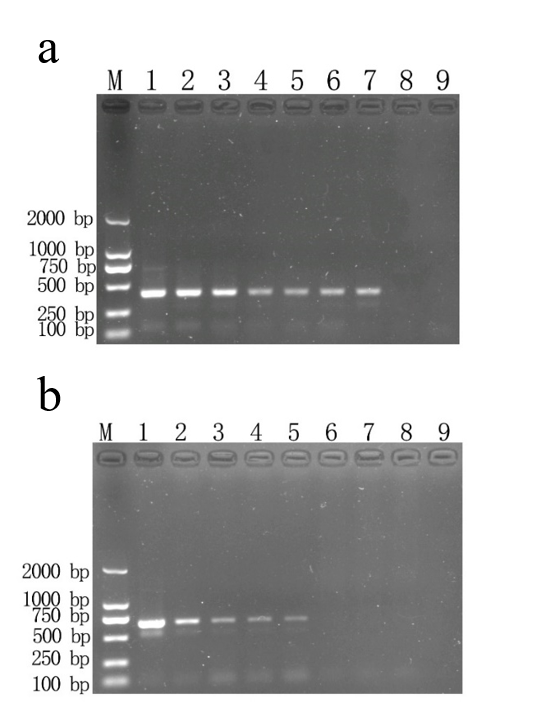


**Figure S6**. Sensitivity of real time qPCR and general PCR for detection of *F. tricinctum.* (a) Amplification plots of RT-qPCR. (b) Melting curve of RT-qPCR. (c) general PCR. M: D2000; 1-8: 31 ng/μL, 3.1 ng/μL, 310 pg/μL, 31 pg/μL, 3.1 pg/μL, 310 fg/μL, 31 fg/μL, 3.1 fg/μL; CK: Negative control. 9: Negative control.


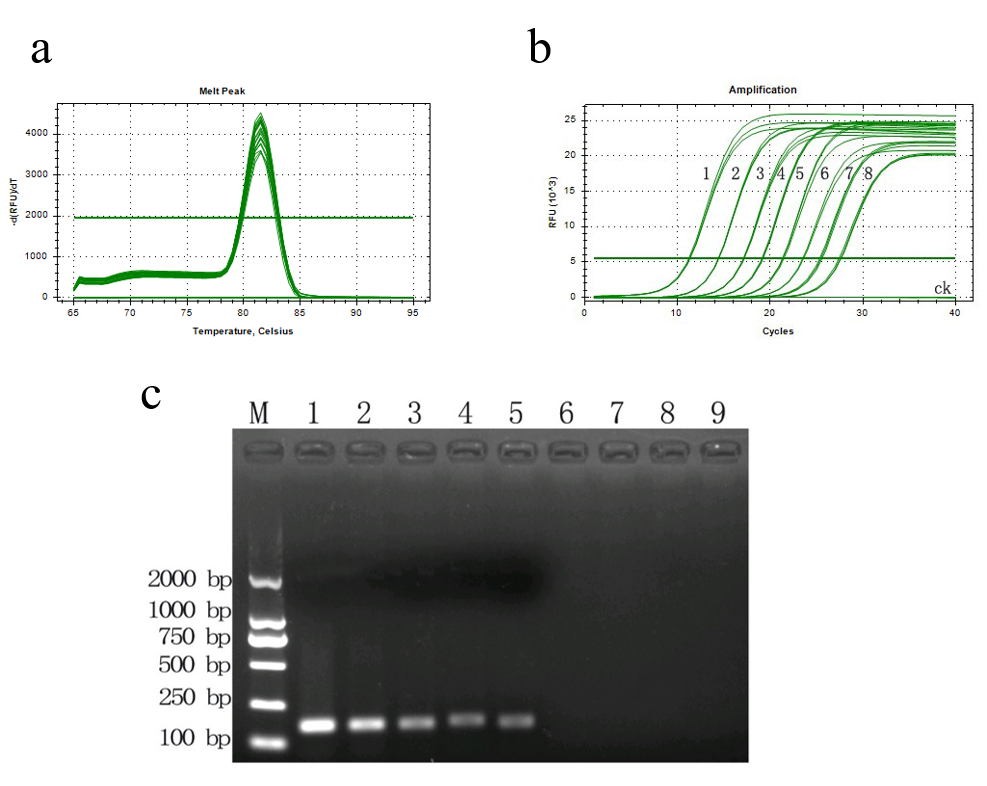

Supplement: Supplementary file 2 — Supplementary Material 2: Table S1. LAMP detection initial reaction system. Table S2. Nested PCR detection initial reaction system. The template for the first round was DNA, and the template for the second round was a tenfold dilution product of the first round of PCR. Table S3. Real Time-qPCR detection system. Table S4. Fungal universal primer sequences. Table S5. Information on the entry numbers of strains used for phylogenetic analysis. Figure S1. The results of LAMP amplification using specific primers. (a) the gel electrophoretogram. (b) Hydroxy naphthol blue (HNB) color rendering. M: D2000; 1-15: numbers as the strain numbers in Table 1, N: negative control. Figure S2. The schematic diagram of the result of nested PCR amplification using specific primers. (a) primer CYP-4F/R. (b) primer C4-10F/R. M: D2000; 1-15: numbers as the strain numbers in Table 1, CK: negative control. Figure S3. A schematic diagram of the result of general PCR amplification using specific primers CP-1F/R. M: D2000; 1-15: numbers as the strain numbers in Table 1, CK: negative control. Figure S4. Sensitivity of LAMP for detection of F. tricinctum. (a) the gel electrophoretogram. (b) Hydroxy naphthol blue (HNB) color rendering. M: D2000; 1-8: 31 ng/μL, 3.1 ng/μL, 310 pg/μL, 31 pg/μL, 3.1 pg/μL, 310 fg/μL, 31 fg/μL, 3.1 fg/μL; 9: Negative control. Figure S5. Sensitivity of nested PCR and general PCR for detection of F. tricinctum. (a) nested PCR. (b) general PCR. M: D2000; 1-8: 31 ng/μL, 3.1 ng/μL, 310 pg/μL, 31 pg/μL, 3.1 pg/μL, 310 fg/μL, 31 fg/μL, 3.1 fg/μL; 9: Negative control. Figure S6. Sensitivity of real time qPCR and general PCR for detection of F. tricinctum. (a) Amplification plots of RT-qPCR. (b) Melting curve of RT-qPCR. (c) general PCR. M: D2000; 1-8: 31 ng/μL, 3.1 ng/μL, 310 pg/μL, 31 pg/μL, 3.1 pg/μL, 310 fg/μL, 31 fg/μL, 3.1 fg/μL; CK: Negative control. 9: Negative control. [file 12866_2025_4295_MOESM2_ESM.docx]
